# The Role of Cell Organelles in Rheumatoid Arthritis with Focus on Exosomes

**DOI:** 10.1186/s12575-021-00158-4

**Published:** 2021-11-04

**Authors:** Zahra Payandeh, Abbas Pirpour Tazehkand, Ali Azargoonjahromi, Faezeh Almasi, Armina Alagheband Bahrami

**Affiliations:** 1 Immunology Research Center, Tabriz University of Medical Sciences, Tabriz, Iran; 2 Department of Biochemistry and Clinical Laboratories, Faculty of Medicine, Tabriz University of Medical Sciences, Tabriz, Iran; 3 Shiraz University of Medical Sciences, Shiraz, Iran; 4 Pharmaceutical Biotechnology Lab, Department of Microbial Biotechnology, School of Biology and Center of Excellence in Phylogeny of Living Organisms, College of Science, University of Tehran, Tehran, Iran; 5 Department of Biotechnology, School of Advanced Technologies in Medicine, Shahid Beheshti University of Medical Sciences, Tehran, Iran

**Keywords:** Rheumatoid arthritis, Exosome, Autoimmune diseases, Therapy

## Abstract

Auto-immune diseases involved at least 25% of the population in wealthy countries. Several factors including genetic, epigenetic, and environmental elements are implicated in development of Rheumatoid Arthritis as an autoimmune disease. Autoantibodies cause synovial inflammation and arthritis, if left untreated or being under continual external stimulation, could result in chronic inflammation, joint injury, and disability. T- and B-cells, signaling molecules, proinflammatory mediators, and synovium-specific targets are among the new therapeutic targets. Exosomes could be employed as therapeutic vectors in the treatment of autoimmune diseases. Herein, the role of cell organelle particularly exosomes in Rheumatoid Arthritis had discussed and some therapeutic applications of exosome highlighted.

## Introduction

What is deemed to culminate in auto-immune diseases is the inability of immune system to distinguish self-cells from non-self-ones. Recognizing the self-cells as foreigner, the immune system attacks and impairs self-molecules. Auto-immunity has been estimated to encompass wealthy nations which accounted for well over 25%, following a swift increase incidence therefrom [1, 2]. Such disorders are prevalent among people being at the age of between 20 and 40. Auto-immune disorders are typically chronic and incapacitating illnesses with a significant medical and financial burden [3–5].

In Rheumatoid Arthritis (RA), aggressive synovial hyperplasia – which destroy articular joints – is a significant hallmark. Several genetic, epigenetic, and environmental factors are held culprits for the initiation and progression of RA [6]. Immune and non-immune cells, non-genetic factors, inflammatory mediators are considered to involve in inflammatory processes which target cartilages and bones, resulting in a noticeable decline in the function of joints. Along with these components, variety range of vulnerable genes, namely human leukocyte antigen (HLA) class II, as well as more than 100 susceptible loci are responsible for reducing joints function. Even though M1 macrophages, T helper 1 (Th1), and Th17 cells have a key role to play in producing pro-inflammatory cytokines, such as Tumor necrosis factor (TNF), interferon gamma (IFNγ), interleukin (IL)-12, − 17, − 18, − 22, − 23, which contribute to the onset of auto-immune diseases, M2 macrophages have the ability to reduce the inflammation and to mitigate the condition via secretion of anti-inflammatory cytokines including IL-4, − 10, − 13, − 35 and transforming growth factor beta (TGF-β) leading [7].

Herein the role of cell organelle such as exosomes in rheumatoid pathogenesis and the therapeutic application of exosomes in RA will be discussed in detail.

### Sundry Phases in RA Pathogenesis

Several aspects have been deemed to exacerbate arthritis, namely genetic, epigenetic, and environmental factors. Environmental risk factors which are included chemicals, smoking, and microorganisms can cause per se local inflammatory responses and immune system induction followed by epigenetic and post translation modifications (PTMs) of proteins [8].

Presentation of native or pseudo-native peptides (in breakdown of tolerance mechanisms) via dendritic cells culminate in T- and B-cells activation, thereafter it produces cytokines and autoantibodies. It was also pointed out that autoantibodies developed during the onset of clinical disease were able to recognize many neoepitopes on the process of epitope spreading [9].

Autoimmune responses to post-translationally both modified and unmodified self-antigens embark upon the disease, prior to emerging subclinical synovitis and clinical symptoms. Furthermore, autoantibodies produced during this preclinical process can cause both bone degradation and pain. Auto-antibodies are attached to various epitopes and create immunological complexes, resulting in synovial inflammation and arthritis [9, 10].

Many tissues are targeted in the RA cases and the main of which is the synovium. Inflammatory and joint-destructive substances are primarily stored in these cells. Auto-antibodies have an important role as mediator of joint inflammation and bone degradation to accelerate the inflammatory processes. Depending on the severity of the disease, auto-antibodies are found in 50–80% of RA patients [11, 12]. These antibodies could activate inflammatory effector pathways in chondrocytes and cartilages, leading to the release of extracellular matrix (ECM) components [13].

In this case, autoantibodies’ glycosylation is crucial. It has been reported that increased IgG-Fc sialylation has been associated to reduce inflammatory bone decay. However, decreased sialylation has been associated to RA and osteoclastogenesis. In short, the main causes of bone degradation are synovial inflammation, pro-inflammatory cytokines, autoantibodies, and receptor activator of nuclear factor B ligand (RANKL) [14]. In the situation in which self-tolerance is disrupted, immune and non-immune cells may be activated, thereby releasing inflammatory mediators. Noteworthy, preosteoclasts can be differentiated at the cartilage-bone interface into bone-resorbing osteoclasts as a result of fibroblasts expressing RANKL and macrophage colony-stimulating factor (M-CSF) [14]. It has been become a prevailing notion that T- and B-cells, signaling molecules, pro-inflammatory mediators, and synovium-specific targets ought to be considered as the novel therapeutic targets [10].

### Role of Different Organelles in RA

#### Mitochondrial in RA

Mitochondrial malfunction has been shown in RA, neurological diseases, diabetes, cancer, and obesity. The linkage between Rheumatoid Arthritis and mitochondrial dysfunction has been shown by numerous studies. Nuclear DNA (nDNA) encodes some proteins that translocated to mitochondria. In the recent times, it has been reported that such proteins have a pivotal role to play in mitochondrial dysfunction, in particularly, in the cases of oxidative stress-related processes, apoptosis, and RA.

The provocation of internal nucleic acid sensors as well as the Toll-like receptor (TLR) 9 are induced by unmethylated CpG patterns in mitochondrial DNA (mtDNA). The immune response of mtDNA is increased by containing 8-oxo-guanine residues. These residues, albeit being far more common in mtDNA than in nDNA, are as a consequence of oxidative events which were caused by reactive oxygen species (ROS) [15]. ROS may arise in the case of RA synovial mitochondria as a result of both the pannus – which increases Adenosine triphosphate (ATP) demand and disrupting microvasculature – which leads to hypoxia through mtDNA, proteins, and lipids serving as initial targets for these free radicals. The pro-inflammatory HIF-1, NF-B, JAK-STAT, AP-1, and Notch pathways are also known to be activated by hypoxia and ROS [15].

In addition to aforementioned, the 1158 nDNA-encoded proteins can be translocated to mitochondria as well as involving in ROS production pathways [16]. In term of pathophysiology, the roles of such proteins in mitochondrial dysfunction and correlation thereof with ROS-mediated, hypoxia, ATP production, and inflammation in the case of RA is considered a vital subject to understand.

It is worth noticing that the apoptosis is important for synovial hyperplasia in RA to be controlled, as it can be triggered by both extrinsic and intrinsic processes. The intrinsic occurs in mitochondria caused by oxidative stress, whereas the extrinsic routs are inactive in the fibroblast-like synoviocytes (FLS) of RA patients. Notably, both mechanisms can lead to the activation of a protease cascade, known as caspases [17].

To recognize importance molecules in cytokine signaling in RA, protein-protein interaction (PPI) networks has been created by some researchers at the cellular level [18]. Importantly related regions, ego networks, and genes in PPI had also pointed out by studies that have link to RA and other illnesses. The PPI was also employed in another study to evaluate the efficacy of the anti-inflammatory medicines, Leflunomide, and Ligustrazine in the treatment of RA [19].

The existence of the electron transport system in mitochondrial has a key role in the modifications of mtDNA. In addition, the absence of histones in mtDNA makes them more vulnerable to ROS induced damages. There are two biochemical variables that influence into immunological capacity. Protein synthesis process in mitochondria is like that of bacteria which begins with a formylated amino acid. Formylated peptides can also stimulate neutrophil receptors, inducing activation and chemotaxis. The other mitochondrial protein – mitochondrial transcription factor A (TFAM) – is immunologically active. TFAM having similar structure to high-mobility group box 1 (HMGB1) can function as an alarmin and promotes inflammation like nucleus counterpart thereof [20].

Some chemicals may leave the mitochondria as permeability breaks down because of cell stress. Cytochrome c will elude the mitochondria’s sinking ship and interact with other molecules, causing apoptosis. In the case that of mtDNA is outside the mitochondria, it can activate the inflammasome by interacting with internal sensors. Owing to sever cell malfunction, all these mitochondrial products leave the cell, and also along with individual components which leak or release, entire mitochondria might depart from the cell with the payload of hazardous molecules. Following the stimulation of eosinophils, the mitochondrial catapult as an intelligent mechanism allows the entire mitochondria to be exteriorized [21].

Platelet activation is the epitome of presenting entire mitochondria in an extracellular position [22]. Microparticles comprised of mitochondria as well as whole mitochondria are found in the microparticles generated after platelet activation. The presence of entire mitochondria in the extracellular space is prominent, showing its similarity to bacteremia occurring during infection. Indeed, this demonstrates the contribution of mitochondria in shock and sterile inflammation [23]. Mitochondrial products having systemic and local functions in chronic autoimmune and inflammatory diseases are involved in the pathogenesis of disorders like RA by promoting synovitis via mtDNA in joints [24].

Terminology is affected by changing personality of mitochondria and the desire for becoming outright hazardous. Relying on the origin of functions, mitochondrial products differ from one another. For instance, products can be DAMPs (disease-associated molecular patterns) and PAMPs (pathogen-associated molecular patterns) if the activity are triggered by cell death and the same molecular structures, respectively [25].

To refrain conflict and prolonged disputes, it is better to use a neutral and non-committal word; to wit: MAMP (mitochondrial-associated molecular pattern) refers to a form of mitochondrial molecular pattern. The discovery of immunological potential of the mitochondrion promotes the increasing picture of molecules which are repurposed to enhance innate immunity [26].

### Endoplasmic Reticulum in RA

The endoplasmic reticulum (ER) biosynthesizes both secretory and membrane proteins. The ER lumen provides a desired situation which is required for proper folding of membrane and secretory proteins. The homeostasis in the ER is preserved by the unfolded protein response (UPR), ER-associated degradation (ERAD), and a structured adaptive program. A variety of factors, such as altered cellular metabolism, mutations in substrate and route chaperones, and infection, are involved in predisposing proteins to misfolding [27, 28].

The initiation of a UPR signaling cascade throughout ER stress – not to mention it is caused by the aggregation of unfolded proteins – is done by the glucose regulated protein 78 kDa (GRP78) which has been known as BiP (binding immunoglobulin protein) [29]. Incidentally, three ER-localized protein sensors, namely double stranded RNA-dependent protein kinase RNA-like endoplasmic reticulum kinase (PERK), inositol-requiring transmembrane kinase-endoribonuclease-1 (IRE1), and activating transcription factor 6 (ATF6) contribute to the progression of the key UPR signaling cascade. The GRP78 impedes PERK, IRE1, and ATF6 to be activated in the resting state through merging into their N-termini. Upon being activated, the GRP78 may be attached to misfolded or unfolded proteins and liberate PERK, IRE1, and ATF6, inducing UPR signaling. The intrinsic ribonuclease activity of IRE1 promotes the formation of X-box binding protein-1 (XBP-1) – a transcription factor which escalates the expression of genes contributing to protein folding and degradation. Inhibition of the phosphorylation of initiation factor 2, nonetheless, can prevent general protein synthesis by PERK [30].

It is worth mentioning that the cell might lose the ability to rectify the protein folding deficiency and restore ER balance under long-term ER stress, thus the activation of cell death programs such as apoptosis and autophagy by the UPR. It has been shown that many diseases such as cancer, ischemia/reperfusion injury, neurological disorders hypoxia, cardiac disease, inflammatory bowel disease, infection, obstructive airway disease, and diabetes are associated with the ER stress response. The impaired ER stress, in turn, can lead to chronic autoimmune inflammatory disorders. The implication of a microarray study, moreover, revealed that the ER response can be escalated in skeletal muscle damage in the situation in which autoimmune myositis as the GRP78 expression is exerted in the muscle tissue of these patients [31].

The impact on RA of ER stress has remained a subject of intense debate. According to recent studies, a linkage between ER stress response and chronic autoimmune inflammation has been reported, whereby ER stress may induce or affect inflammatory disorders’ phenotype. Likewise, a study carried out to realize the impact of GRP78 on RA pathogenesis reached meaningful consequences. In the inflamed RA joints, some conditions are capable of increasing ER stress in both innate and adaptive immune cells; to wit: hypoxia, ROS, glucose deprivation, and pro-inflammatory cytokines. Inasmuch as the expression of GRP78 in RA-FLS is particularly upregulated in response to ER stress, such event enhances FLS survival and proliferation, hence synovial proliferation. The level of ER stress-mediated GRP78 in the ER lumen increased then can re-localize to cell surface from the ER, which in turn can be considered a target for Anti-citrullinated protein antibodies (ACPAs) and act as an auto-antigen for T- and B-cells [32]. Furthermore, extracellular GRP78 found highly in RA joints can promote the production of IL-17 and TNF in RA synovial mononuclear cells, and also it increases the growth of auto-reactive T-cells.

In addition, having bound to ACPA [33], citrullinated GRP78 on monocytes/macrophages escalates GRP78 expression in RA-FLS by promoting the production of pro-inflammatory cytokines, such as TNF α. This event per se can lead to amplifying the inflammatory cascade via promoting the formation of pannus [34]. Eventually, GRP78 promotes vascular endothelial growth factor (VEGF)-induced migration/chemotaxis and the endothelial cells proliferation as well as stimulating synovial angiogenesis, and also it has been considered as a vital ER chaperone [35, 36].

According to several studies carried out to ascertain other functions of GRP78, they pointed out that both cytoplasm and cell membrane are encompassed of GRP78, and also it plays a remarkable role in cell survival, metastasis, tumor angiogenesis, and resistance to chemotherapy. The discovery that GRP78 exists on FLS surface can pave the way to novel therapeutic means targeting the pathologic hallmarks of RA, synoviocyte proliferation, and endothelial cells [37].

The conjunction of toxin or apoptosis-inducing with the synthetic peptides having the potential to be blended with GRP78 such as WIFPWIQL peptide, may suppress synovial angiogenesis, proliferation, and pannus development [38]. It is important to note that since extracellular GRP78 induces T cell tolerance and rivals with membrane GRP78 to bind with the anti-GRP78 antibody, it can decrease RA activity.

Although RA-FLS studies, focusing mainly on changing occurred in cellular viability and possible repercussions for synovial hyperplasia thereof, it was myeloid-specific manipulation of UPR pathways that was shown to culminate in a remarkable decline in cytokine expression and in reducing K/BxN serum-induced arthritis. It was also pointed out that ER stress condition affect the RA-FLS to resist apoptosis, owing to increasing the rate of autophagy and the activation of proteasoms. Even so, our knowledge is limited of how ER stress has impact on FLS ability to influence synovial inflammation.

In recent times, a linkage between XBP1 splicing and TLR-mediated activation of RA-FLS has been reported [39]. Noteworthy, the presence of a significant ER stress signature is a distinguishing hallmark of RA synovium, as the IRE1α-XBP1 axis in RA synovial tissue is conducive to macrophage responding to TLR signaling in the condition being devoid of ER stress. Nonetheless, TLR stimulation increases cytokine and chemokine production in the cases of presence ER stress in stromal cells. Despite what has just been mentioned, how ER stress condition influences pathogenic processes has remained unknown [40].

### Cytoskeleton in RA

The actin cytoskeleton fulfills many functions such as cellular homeostasis, modification of cell shape – migration, differentiation, and development thereof. It also has pivotal role in wound healing, polarity maintenance. To accomplish adequately such duties, it is imperative for every cell to coordinate its actin cytoskeleton with all external and intracellular. For instance, the number of both filamentous F-actin against monomeric G-actin and stress fibers are necessity to be controlled so as to movement. To this end, a complex intracellular protein network must be built, thereby allowing the cell to control actin polymerization, nucleation, depolymerisation, and actin organization relying on the cell’s needs [41].

Modulation of actin organization as well as controlling actin polymerization is of great importance. As a result, variety of signaling and/or actin-binding proteins can alter the actin cytoskeleton structure, leading to focal adhesion, stress fibres, lamellipodia, or even filopodia formation based on the needs of specific cells at the given time [41–43]. Notably, tiny GTPases of the Rho family proteins – Rho, Rac1, and Cdc – belonging to the Ras protein family and having been found in sundry isoforms, modulate the formation of projections in the membrane enclosing the cell. These proteins are able to bind with GTP and to impart an active GTP-bound form to an inactive GDP-bound form on a regular basis. The other function of such proteins was shown to interact with several effector molecules in the active state to deliver a specific signal. Rac1 and Cdc42 mediate the production of lamellipodia and filopodia if they be activated by growth factors and integrins, respectively [44, 45].

Nevertheless, it has been discovered that the activation of TNFα-induced NF-kB and cytokines secretion depends on the activation of RhoA in human cultured rheumatoid arthritis synovial fibroblasts (RASFs), which in turn this implies a crucial function of this GTPase in the arthritic inflammatory response. The implication of this issue is that the development of stress fibres can be exerted by Rho. Moreover, it has been pointed oud that the depletion of gelsolin – an actin binding protein involved in actin depolymerization by preventing F actin production – has exacerbated the illness in mice, resulting in the importance of gelsolin in regulating the actin cytoskeleton in RASFs of the RA cases [46, 47].

According to a recent research on cadherin-11-deficient mice, the importance of tissue remodeling in RA was highlighted. The implication of that was to realize an important role of RASFs in reducing the migration, invasion, and adhesion capabilities. Whereas the resistance to the K/B6N serum-transfer arthritis (STA) was shown to be accomplished by RASFs [48, 49].

In addition to aforementioned, the actin cytoskeleton has noticeable impact on cellular morphology and gene transcription, hence activating Mal – a modulator of gene expression. Following F-actin formation, Mal is separated from G actin, thenceforth it stimulates the serum response factor (SRF) as well as target genes which include genes controlling proliferation, cell growth, differentiation, and the actin cytoskeleton machinery. Interestingly, a dynamic relationship between actin cytoskeleton remodeling and SRF was found, as the ablation of the SRF can cause low F-actin levels and remarkable decline in migration and adhesion abilities in murine embryonic stem cells [50, 51]. Furthermore, increased F-actin synthesis may cause increased gene expressions which are mediated by SRF and NF-kB, leading to the maintenance of the disorganized actin cytoskeleton and finally to the generation of pro-inflammatory cytokines. All this event holds the arthritogenic response’s negative effects [50, 51].

More importantly, the actin cytoskeleton in numerous disease states, aside from RA, has the ability to maintain cell homeostasis and survival as well as playing significant role to keep going the function of many proteins being essential to fine-tune their organization. And also, a linkage was revealed between the failure in actin collection and polymerization in polymorph nuclear leucocytes with recessively inherited genetic condition known as neutrophil actin dysfunction. The actin polymerization is therefore important for neutrophil to move [52].

What has been found to be increased among patients suffering from RA is the serum amyloid level; this elevation results in synovial hyperplasia and angiogenesis. Actin and/or actin-binding protein-associated diseases have been linked to affect cancer cells, as they can affect tumor cells to progress and to metastasize [53]. Incidentally, various changes occurring during the metastatic process and tumor progression are as a result of deregulating the actin-binding proteins and actin cytoskeleton at either cellular matrix or cell–cell adhesion sites – not to mention such event happens in RA as well [52]. To clarify to understand by an example, diminishing expression of E-cadherin, deemed a cell–cell adhesion molecule, has caused poor prognosis and cancer progression. Deregulation of many ECM adhesion proteins, irrespective of having direct or indirect association with actin, play key roles in tumor progression and metastasis by either altering cell proliferation or generating anoikis – which is a type of apoptosis highlighted by the loss of ECM contact and adhesion [54, 55]. Notably, it has been recommended that some of which be considered as potential targets for novel anticancer therapeutic methods [43]. To achieve this goal that the actin cytoskeleton can be considered as novel therapeutic method, the suppression of signaling inducing actin polymerization by either short peptide compounds or direct interference with their gene expression can be an effective way.

Antikeratin antibodies have been found among nearly 68% of patients suffering from rheumatoid arthritis hand abnormalities, reaching a prevailing notion that there is an important linkage between Antikeratin antibodies and RA [56]. Since Antikeratin antibodies act against citrulline, actin and myosin may become a target for anti-cytoskeletal antibodies upon citrullination. Some proteins citrullinated can induce this process, too. In short, patients afflicted by rheumatoid arthritis were shown to have higher amount of autoantibodies acting opposite to cytoskeletal antigens, particularly the myofibrillar proteins actin and myosin [57, 58].

### Exosome and Extracellular Vesicles in RA

Exosomes are nanoscale cell-derived extracellular vesicles (EVs) with 30–100 nm in diameters [59]. They can be generated by the reverse budding of multivesicular bodies and extricated when they fuse with the plasma membrane. Exosomes were originally assumed to be used for removing unwanted proteins from cells and were named “trash cans” [60]. Later, they were used to deliver bioactive compounds, such as proteins and microRNAs, in intercellular communications, called cytosol gulps [61].

Exosomes fulfills various function as well as having noticeable roles in sundry processes irrespective of type of cells. The most important of such functions has been noticed is to transmit bioactive molecules among cells. Along whit transmitting, some conditions such as transmissible spongiform encephalopathies, Alzheimer, Parkinson, and amyotrophic lateral sclerosis can be affected by exosomes [62–64]. Exosomes have been reported to be shed by both immune cells, namely mesenchymal stem cells (MSCs), mast cells, lymphocytes, and dendritic cells, and even notably tumor cells [65, 66].

Depending on conditions and correlations, exosomes can deliver different substances. As an example, exosomes contained the TCR/CD3/zeta complex and major histocompatibility complex class II (MHC II)-peptide can be shown in the situation in which T-cells correlate with antigen presenting cells (APCs) [67]. Additionally, MSCs increase remarkably releasing exosomes bearing anti-inflammatory molecules which are imperative for polarizing macrophages into the M2 phenotype in the case of hypoxia preconditioning and in the presence of lipopolysaccharide [68].

Dependent and independent fusions have been considered as two different methods accomplished by exosomes to influence into nearby cells. Fusions per se are divided into two categories: 1) direct fusion pertaining to plasma membrane of the recipient cell – which relies on receptor-ligand interactions, 2) Back fusion relating to the vesicles are undergone endocytosis by recipient cell and hence the inclusion of the vesicles into the endosome’s membrane. However, the inverse function is shown in the fusions encompassed independent method, as the interaction between vesicles and recipient cells is incident. Function of delivering MHC-peptide having link to the T-cell receptor (TCR) on T-cells fulfilled by exosomes is the quintessential of such fusion [69, 70]. To identify exosomes, it can be done by different aspects, such as exosome-specific membrane proteins, cell types, condition-specific proteins, genetic materials, mRNA, and miRNA.

Long noncoding RNAs (lncRNAs) has been become a demand subject to investigate since it has impact on embryonic differentiation, pluripotency, and development. It was also reported to play key roles in variety of illnesses [71]. Serum exosomal Hotair – HOX transcript antisense RNA found as the first lncRNA – was shown to correlate highly with clinical features of laryngeal squamous cell carcinoma (LSCC), particularly in the situation in which the level of its serum expression is evaluated. That is why it can be used as a biomarker to monitor LSCC and be considered as a prediction tool for patients afflicted by LSCC. What is worth mentioning is that it has been discovered among the exosomes of patients suffering from RA in the case of high expression level of Hotair, culminating in promoting macrophage migration [72]. Conversely, the amount of Hotair among some disorders including differentiated osteoclasts and rheumatoid synoviocytes has been shown negligible. This issue induces the expression of Hotair, thereby much lower level of matrix metalloproteinase (MMP)-2 and MMP-13 as well. Although more researches is imperative to reach a prevailing notion in terms of making use of exosome-derived Hotair so as to sundry subjects, it can be deemed as a potent biomarker to diagnose RA [73].

### Mechanism of Exosome Function

Exosomes are thought to be vehicles delivering immunosuppressive substances generated from their parents’ dendritic cells (DCs), even though the precise functional mechanism of immunosuppressive DC-derived exosomes has remained thus far unknown [74].

To exert suppressive molecules their duties, the presence of certain substances on both exosomes and recipient animals is necessity; MHC II and B7–1/2 molecules are the epitome of such substances.

According to trafficking analysis, exosomes cross-trafficking to the contrariwise lymph node is limited. Immunosuppressive DC exosomes therefore can change the function of endogenous immune cells, such as APCs, culminating in a systemic suppressive/anti-inflammatory impact [75, 76]. Interaction between exosomes and APCs may also be shown in some situations, namely the membrane level, via the internalization of such vesicles, hence providing a situation for both vesicle-contained proteins and RNAs to transfer effectively [74].

### Mesenchymal Stem Cell-Derived Exosomes

Friedenstein et al. elicited MSCs from guinea-pig bone marrow and spleen. MSCs are deemed as self-renewable, multipotent, and spindle shape cells [77]. Both pericytes and MSCs coming from common root have adverse impact on CD45, CD34, and CD56 as well as expressing CD29, CD73, CD44, CD105, CD90, and CD146 [78]. MSCs are classified in accordance with the capability of which to produce chemicals to enhance both regenerative and immunoregulatory functions. These cells can have link to cells in vitro, cells transplanted, and repairing the damages [79]. Under normal conditions, differentiation of MSCs into other lineages is not shown in vivo. The function of MSCs, Nonetheless, is inverse in detrimental situations, releasing regenerative and immunoregulatory factors. MSCs are capable of ascertaining injurious and inflammation conditions via dynamic immunomodulatory profile, followed by switching to the required response [80].

Nitric oxide (NO), TGF-β, soluble HLA-G, indoleamine 2,3-dioxygenase (IDO), and prostaglandin E2 (PGE2) can be produced by MSCs as a result of responding to high concentrations of TNF α, IFN γ, and TLR3 agonists. This reaction, even though activating CD4 + CD25 hiFoxP3+ regulatory T-cells (Tregs), can suppress natural killer (Nk) and T-cell responses [81, 82]. Exosomes, as well as direct secretions of soluble molecules, are responsible for delivering such components.

In addition to what has been cited, cytokines affected by MSCs are the other molecules having impact on immune system. IFN – which is affected by MSC-derived cytokines/growth factors – as well as other cytokines such as TNF α, IL-1ß, IL-1, and IL-17 have been considered as immune modulator [83]. In addition, IFN provokes the production of molecules to regulate immune response; these molecules are vascular cell adhesion molecule 1 (VCAM-I), Jagged-1 and 2, intercellular adhesion molecule 1 (ICMA-1), programmed death-ligand 1 (PD-L1), and HLA G1. Other immunomodulatory molecules also can be released by MSCs, namely IL-10, TGF, hepatocyte growth factor (HGF), galectin (Gal)-1, IL-6, IDO and NO to modulate innate and adaptive immunological responses [83]. On the whole, MSCs accomplish various actions, namely inhibition of T-cell proliferation and cytokine secretion, regulation of Th1/Th2 balance, controlling Tregs, and regulation of B-cell activity and DCs antigen presentation [84].

### Mesenchymal Stem Cell-Based Therapy for Autoimmune Diseases and RA

Interestingly, it has been recommended that MSCs be a unique alternative therapeutic for autoimmune illnesses because MSCs were shown to have a significant immune-regulatory impact against autoimmune illnesses. Further to this issue, MSCs were shown to be able to reduce the proliferation and activity of NK cells, as well as T/B-cells proliferation and DCs maturation. MSCs have also sparked a lot of interest for usage in autoimmune illnesses due to these therapeutically relevant characteristics [85]. As to recent findings, MSCs positive effects in autoimmune illnesses are based on both direct cell-to-cell interaction and MSCs paracrine function [86].

However, the percentage of culture-expanded MSCs may be survived and be integrated with host tissues is negligible, accounting for less than 1 %, thereby not being found meaningful therapeutic impacts of MSCs by direct cell-to-cell interactions [87]. Most importantly, secretory growth factors and extracellular vesicles which produces vast range of biomolecules – proteins, mRNA, and microRNAs – can induce MSC-based therapy so as to many diseases, namely autoimmune illnesses [88]. The EVs as well as MSCs enhance myriad physiological tasks, as the most important of which can be included genetic exchanges, cell proliferation and differentiation, antigen presentation, tumor metastasis, angiogenesis, and immune system responses [89]. The capacity of MSC-derived EVs to imitate the impacts of the original cell on a variety of effector cells has also been investigated. MSC-derived EVs are involved in the repairing damaged tissue and in the modulation of immune system, they never represent the drawbacks of their parental cells but still and all [89].

The immunosuppressive properties of MSCs can be a result of TGF-β synthesis by these cells. TGF-β1 produced by MSCs controls the activity of mast cells, NK cells, macrophages, T-cells, and microglia [90]. MSC-derived TGF1 produced is also involved in the enhancement of T helper (Th) subsets. Overexpression of TGF1 in mBM-MSCs, for example, was demonstrated to improve their therapeutic potential in type 1 diabetes model with an enhanced Th2 response [91, 92].

Since the impact of MSCs on B cells has been considered a double-edge sword effect, this issue has been remained a subject of intense debate. To clarify by an example, they, on the one side, have the capability to decrease B-cell proliferation and antibody generation if they be exposed to IL40, cytosine–phosphate–guanosine (CpG), CD40L, anti-immunoglobulin, interleukin (IL)-2, and IL-4. But on the other side, MSCs were shown to not have effect on B cells in the situation of that they are provoked by allogeneic T-cell-depleted peripheral blood mononuclear cells (PBMCs) and CpG [93].

When it comes to the impact on other immune cells of MSCs, they downregulate MHC II and co-stimulatory molecules expression, thus influencing into DC development. MSCs also affect the expression of IL-10 and IL-12 [94]. Under the condition of high expression level of TGF-γ, MSCs diminish NK cell proliferation and increase Treg lymphocyte differentiation [95].

MSCs have been shown to prevent infection by delaying neutrophil death. MSC-derived exosomes, like their parental cells, are generally hypo-immunogenic, with the lack of MHC-II and low expression of MHC I. MSCs, furthermore, have been shown to produce more exosomes than human cell lines such as the human embryonic kidney (HEK) and human acute monocytes leukemia (THP-1), reaching to such deduction that MSCs can be deemed as the most useful source of immunoregulatory exosomes [96].

Cosenza et al. compared the functions of cryopreserved MSC-derived exosomes to those of newly obtained exosomes and found that preservation destabilizes the membrane’s integrity, resulting in vesicle aggregation, content leakage, and a reduction in immunosuppressive capacity [97]. MSC-derived exosomes clearly have both MSC markers like CD90, CD44, and CD73, as well as exosome markers including CD63, CD9, and CD81 [97]. These markers are considered to be used to track exosomes utilized to deliver drugs. Exosomes were predominantly discovered in the spleen, liver, kidney, and lung, known as filtering organs of the body [98]. As lower sensitivity of fluorophores can be used to chase organ localization of EVs, the impact of which on pharmacokinetic analysis of their applications was demonstrated, particularly in terms of the quantity of exosomes collected in a specific target tissue [99]. Several methods were demonstrated to detect sensitivity and to take profs by imaging of animals that are being treated by exosomes. The most noticeable of which is Iodine-125 radiolabeling and bioluminescence generated by luciferase. MSC-derived exosomes, Even though being the target of many investigations to achieve this goal to use them as immune-modulator , the impact of such tiny vesicles on autoimmune disease treatment has hitherto remained in infancy thereof [100].

To manage the symptoms of RA, a variety of biological disease-modifying anti-rheumatic drugs (bDMARDs) are available. For many years, inhibition of proinflammatory cytokines such as GM-CSF, TNF, and IL-6 using antibodies or soluble decoy receptors has been the mainstay of treatment for individuals suffering from RA [101]. Immunosuppressive and immunomodulatory medicines have sundry side effects, including liver issues, nausea, an increased risk of infection, lymphopenia, and so forth [97], so new therapy modules having fewer side effects are highly demanded, namely MSC-derived exosomes due to supporting its immunosuppressive properties in rheumatic diseases. Although the usage of exosomes obtained from a variety of sources has been extensively investigated, the studies carried out into the function of MSC-derived exosomes are still in their early stages [97]. Despite what has been cited, having anti-inflammatory impact of MSC-derived exosomes on T and B cells in a mouse model of collagen-induced arthritis (CIA) has been reported [102].

Other roles of MSC-derived exosomes found are to suppress T-cell proliferation in a dose-dependent manner and to decline the amount of mature T- and B-cell subsets [103]. Notably, exosomes more effectively enhance the population of Tregs than MSCs and MSC-derived microvesicles. It is important to be mentioned that microvesicles derived from MSCs in accordance with recent studies are less effectiveness to induce TGF-β and IL-10 production in B- and T-cells than MSCs do such function alone [103].

According to a study carried out in 2018 by Chen et al., the impact of miRNAs on reducing recruitment and invasion of FLS was investigated. They found that miR-150 carried modified MSC-derived exosomes targets MMP-14, thus diminishing invasion of FLS [103]. In addition, the injecting of such modified exosomes into CIA mice can reduce the thickness of the hind paws and the arthritis scores among animals. Other finding was a study carried out on the effect of human umbilical cord mesenchymal stem cells (hUCMSC)-derived exosomes on progression of RA. The implication of that was an notion that hUCMSC-derived exosomes culminate in decreasing the recruitment of inflammatory cell to the joints and in protecting against joint synovial hyperplasia in CIA rats [104].

Osteoprotegerin (OPG) is central for modulating bone repair and regeneration. The authors hypothesized that the underlying process could be linked to the regulation of the RANKL/OPG imbalance. They found that RANKL levels in the CIA rats’ serum and synovial tissues were reduced while the therapy increased OPG levels. In line with this study, hUCMSC derived exosomes injected can decline autoreactive function in filtering chemokine ligand CCL2 and CXCL12 in both blood and synovial fluid [105].

### Dendritic Cells Derived Exosomes

Exosomes can be produced in large quantities by most of hematopoietic cells, including DCs. DCs are professional APCs which are generated from CD34+ stem cells with capability of regulating immunological reactivity. Although DCs were once thought to cause immunological responses such as organ graft rejection and autoimmune diseases, they have been associated with the progressing and maintaining tolerance to allo/auto antigens among tentative animals [106].

Several factors influence DCs ability either to promote or to repress immune responses, the most important of which are their differentiation/maturation/activation stage and hematopoietic lineage affiliation. Noteworthy, antigen-specific T-cell responses can be reduced by immature DCs (iDCs), because of low levels of MHC and costimulatory molecules such as CD86, CD80, ICAM-1, CD40, and ICOSL. Incidentally, such “tolerogenic” DCs reduce amount of type 1 cytokines – IL-12 family member – and increase the level of immunosuppressive cytokines, namely TGF, IL-10, and VEGF. They also are found at the great quantities into the tryptophan catabolizing enzyme IDO, whereby they destroy free tryptophan and “starves” responder T-cells. This function, in turn, can lead to an increased T-cell death event [107].

Induction of Treg, production of antigen-specific T-cell anergy or deletion, and polarizing T-cells are considered the other epitomes of processes regulated by tolerogenic DCs.

DCs can be ameliorated by both genetic manipulation and exposure to cytokines or even to cytokine inhibitors, thereby stabilizing immunosuppressive/tolerogenic characteristics thereof [108]. Additionally, genetically engineered DCs were shown to have impact on mitigating symptoms and on regulating disease development in the case of autoimmune diseases such as RA and type 1 diabetes. DCs transduced by viral vectors producing immunosuppressive drugs have been shown to be more efficient in treating mouse CIA than similarly modified fibroblasts or T-cells [109].

Exosomes generated from pure DC are exceptionally stable vesicles that can represent a future treatment option for arthritis and other autoimmune diseases. Indeed, these vesicles can be employed as therapeutic vectors in the treatment of autoimmune illnesses. Exosomes derived from iDCs and being relevant to IL-4 and IL-10 immunomodulatory cytokines, are able to diminish the severity of established CIA in mice and to inhibit inflammation in a murine footpad model of delayed-type hypersensitivity (DTH) [110]. Therefore, because of having been deemed DCs as carriers to impart immunosuppressive cytokines in CIA mice model [111], exosomes can be useful therapeutic option to be used to treat autoimmune illnesses like RA rather than DCs.

Other advantages of exosome-based therapy in comparison with gene and cell therapies are included the immunoregulatory or tolerogenic feature of exosomes as well as being situated in blood plasma and serum, providing a novel and safe treatment approach for arthritis [112, 113].

Thus, although exosomes originated from immunosuppressive DCs have robust and long-lasting immunosuppressive effects, such exosomes can be better than their parent DCs since the high efficacy of therapeutic results therefrom has been shown to treat animal DTH and CIA, hence being potential for clinical application in RA [114].

What has made the DC-derived exosomes remarkably safe in vivo delivery is the most important feature of which that they are more stable after isolation, compared to autologous cells. However, it ought to not be forgotten that DCs altered ex vivo have remained hitherto susceptible to maturation in an inflammatory environment. This issue is Because exosomes have a longer half-life than many other cell types following injection [74].

Exosomes elicited directly from ACS seemed to mitigate the symptoms of patients afflicted by RA, making this idea that immunosuppressive exosomes be used in the clinic [115]. On the other side of this idea is the other features of exosomes that they have heterogeneous biological origins and are poorly defined composition, in spite of being safe, feasible, and effective to have splendid outcome [115]. To reach a prevailing idea so as to making use of such exosomes to treat these diseases, further investigation is imperative to be carried out. Altogether, there are firm evidence showing that immunosuppressive exosomes can manage the immune system over reactivity.

### DC/IL-10 Exosomes

Along with anti-inflammatory effects and reduced mice CIA detected in DCs treated with recombinant murine IL-10 (rm.IL-10) or adenoviral IL-10 gene (ad.IL-10), the released exosomes by DCs show immunosuppressive activities [110]. In a mixed lymphocyte reaction, exosomes generated from ad.IL-10 DCs can decrease the proliferation of T-cells. More importantly, local injection of adIL-10treated-DCs or their exosomes suppressed the keyhole limpet hemocyanin (KLH)-induced DTH reaction in mice. It is worth noticing that a single dosage of these exosomes via systematically administered after the onset of CIA increases the illness progression, whereby DC exosomes are more appealing therapeutic than DCs. The suppressive action of DC/IL-10 exosomes is not mainly related to delivery of the inhibitory IL-10 cytokine, though it is not still fully elucidated [110].

Considering the therapeutic impact of exosome depends on the exosome membrane stability and integrity. For instance, repetitious freeze-thaw cycles disrupt the exosome structure, eliminating its curative effects. Exosomes lacking MHC II were shown to not be able to decrease DTH, indicating its critical role in cell-mediated immunity. Besides, B7–1/2 (CD80 and CD86) are necessary for the suppressive effects of IL-10-treated BMDC-derived exosomes. On the whole, the modified DC-derived exosomes can be more immunosuppressive in IL-10 based therapies [111].

### DC/IL-4 Exosomes

DCs undergone genetic modifiers which express IL-4 cytokine were shown to be a successful therapeutic candidate for murine CIA. As to a study, CIA mice were given immature bone marrow-derived dendritic cells (BMDCs) infected with an IL-4 expressing vector by an intravenous injection, thereby relatively full disease suppression over at least 4 weeks. According to another report, the therapeutic impact of IL-4 expressing DC (DC/IL-4) was much higher than rm.IL-4 or ad.IL-4 injection [116]. For example, after a single i.p. injection of IL-4–transduced DCs, the prevalence and severity of CIA were diminished [109].

Exosomes derived from DCs systemically and locally injected were observed to diminish intensity of CIA, and inhibit the DTH response, respectively. The immunosuppressive effects of DC/IL-4 are the quintessential of this finding. To suppress the DTH response, merely syngeneic DC exosomes – not allogeneic ones – were effective. And also, it was syngeneic DC exosomes that was able to move to the spleen and liver, interacting with CD11c + DCs and F4/80+ macrophages [117]. In the DTH model, adoptive transfer of CD11c + or CD3+ splenic cells isolated from systematically-immunized mice with exosomes into the recipient mice’s footpad dramatically were found to reduce footpad edema, demonstrating that DC/IL-4 exosomes can change the function of endogenous APCs and T-cells directly or indirectly either. It can be exerted by either activating the regulation of subset and/or depleting antigen-reactive Th1 cells [117].

### DC/Death Ligand Exosomes

To alter strategy of downregulation of antigen-specific T-cell responses, APCs expressing death ligands e.g., Fas ligand (FasL) and TNF-related apoptosis-inducing ligand (TRAIL) undergone genetic engineered mechanisms can be considered a useful alternative. It is because of their function pertaining to selectively inducing apoptosis of antigen-specific T-cells [118]. The other function of which is worth to mention is to decline donor-specific T-cell hyporesponsiveness to alloantigen and to lengthen the allograft survival.

Depending on systemic and local injection, the results of them differ from one another. For instance, systemic injection exosomes, FasL-expressing DCs decrease the collagen-reactive T lymphocytes, and the course of murine CIA [116], whereas local ones were shown to have an anti-inflammatory impact among the mouse DTH model [76]. Notably, gene transferring FasL to DCs is able to restore the immunosuppressive impacts of FasL-deficient DC exosomes. Noteworthy, treatment process of murine CIA by systemic injection of DC/FasL exosomes was seen flourishing [76].

It is demonstrated that the inhibitory impact was dependent on MHC II – not MHC I making use of DC and not exosomes derived DC from various knockout mice models. Further to a comparison of synovial fluid acquired from patients suffering RA with traumatic arthritis patients, a remarkable linkage between bioactive APO2 ligand (APO2L)/TRAIL – deemed a TNF family member being able to cause cell apoptosis – with exosomes was found. APO2L/TRAIL can also make invading T-cells within the synovial fluid of patients suffering RA by far sensitive to apoptosis [119].

Furthermore, in a rabbit model of RA, bioactive APO2L/TRAIL attached to the membrane of liposomes, was shown to significantly reduce inflammation following intra-articular injection, outperforming soluble, and unconjugated APO2L/TRAIL. Increasing cross-linking receptors as a consequence of higher local protein concentrated upon liposome distribution can elucidate the increased bioactivity [120].

### DC/IDO Exosomes

Decreasing T-cell activation and responses to auto- and allo-antigens can be induced by IDO during tryptophan depletion and/or producing toxic metabolites, as immunoregulatory DCs express the tryptophan catabolic enzyme [121, 122]. In DTH and CIA models, both BMDCs transduced adenovirally producing IDO and DC/IDO exosomes display anti-inflammatory effects. Moreover, transducing DCs by the IDO inducer Cytotoxic T-lymphocyte-associated Protein 4 (CTLA4)-Ig can result in IDO induction and in enabling the exosomes attenuate inflammation [123].

Under situation in which DCs be pre-treated by either the IDO inhibitor – 1-MT – or high L-tryptophan, the suppressive impact of DC/CTLA4-Ig exosomes is shown to be diminished. The implication of such event is that the potential function of DC/CTLA4-Ig mainly relies on the activation of IDO in DCs and on deprivation of IDO-mediated tryptophan. The other molecules having significant role in provoking DC/IDO exosomes to play as an immunosuppressive substance is B7–1/2 molecules. Exosomes obtained from DC/IL-10, for instance, play an impaired anti-inflammatory role if they be transduced by IDO [123].

## Conclusion

Autoimmunity are distinct for each rheumatic disorder of that is described by the existence of disease-specific or disease-associated antibody profiles. RA is a multifactorial disorder, as the initiation and progression of which are affected by several factors, namely environmental and serological. Genomic technologies and computational techniques have paved the way for understanding mitochondrial dysfunction in disorders including RA, obesity, cardiovascular disorders, Leigh syndrome. Furthermore, metabolic profiling and whole-exome sequencing have firmly been associated with both mitochondrial metabolic abnormalities and bi-allelic mutations – which cause mito-ribosomal subunit instability.

The present therapies are being used in the case of RA – biological therapies including proteins and antibodies associated with inflammatory factors – are mainly focused on relieving the symptoms of the disease rather than on reversing disease. Although novel therapy methods pertaining to genes have been deemed to promise to treat such disorders, the standpoint of that whether such approaches are safe enough has remained a subject of controversy. With regard to several studies, it has been explicit that in the case of animal models inflammation as well as autoimmune diseases such as RA, exosomes derived from immunosuppressive DCs and blood plasma and serum can act beneficial functions to enhance treatment processes.

In addition to aforementioned, MSC-derived exosomes can affect cartilage and synoviocyte in rheumatic diseases. These effects are included inhibiting cartilage to be degraded, rather promoting regeneration thereof via anti-fibrotic, anti-apoptotic, and immune-modulatory impacts. They suppress synoviocyte proliferation, too.

**Fig. 1:**
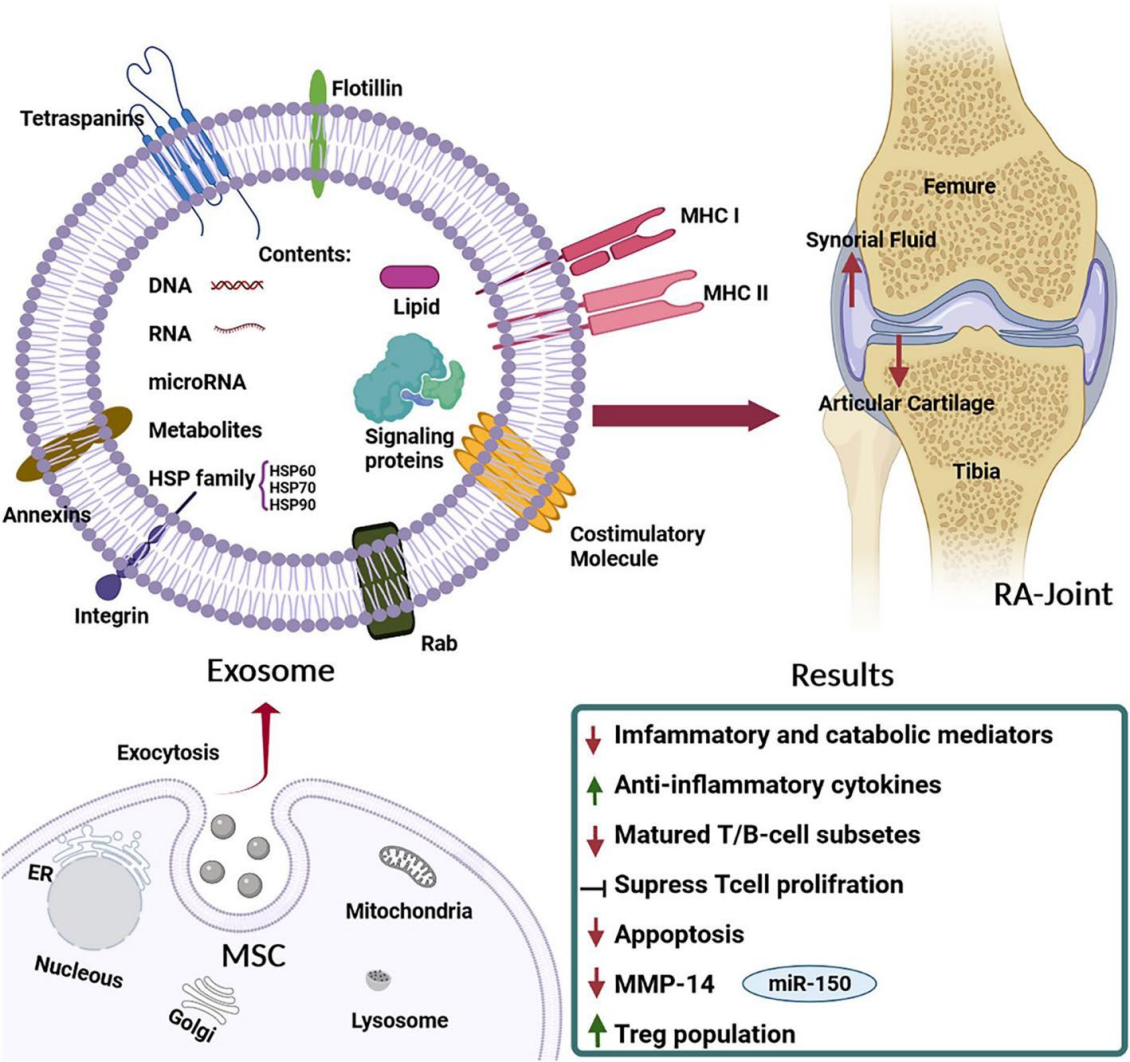
A schematic representation of exosome therapy. Application of exosome in the treatment of Rheumatoid Arthritis. MSCs-derived exosomes can act as immunosuppressive particles having prohibitive and regulatory impacts on both adaptive and innate immunity, whereby they can inhibit bone erosion and promote joint tissue repair

It also is pivotal to be noted that even though DC-derived exosomes can act as immune-modulator, thereby reducing RA pathogenesis via their anti-inflammatory features, other immune-modulatory exosomes – TNF-bound ones – were shown to act as a provocative for pro-inflammatory results, thus exacerbating RA pathogenesis. On the whole, making use of exosomes can be deemed to be effective, unique, and safe therapeutic method so as to the treatment of arthritis. A schematic representation of exosome therapy for RA joints showed in Fig. 1.

## Data Availability

All data and materials are within the paper.

## Abbreviations

ACPA: Anti-citrullinated protein antibody
APCs: Antigen-presenting cells
Apo2L/TRAIL: Apo2 ligand/tumor necrosis factor-related apoptosis-inducing ligand
ATF6: Activating transcription factor 6
ATP: Adenosine triphosphate
bDMARDs: Biological disease-modifying anti-rheumatic drugs
BiP: Binding immunoglobulin protein
BMDCs: Bone marrow-derived dendritic cells
CD: Cluster of differentiation
CIA: Collagen induced arthritis
CpG: Cytosine–phosphate–guanosine
CTLA4: Cytotoxic T-lymphocyte-associated Protein 4
CXCL: Chemokine ligand
DAMPs: Disease-associated molecular patterns
DCs: Dendritic cells
DTH: Delayed-type hypersensitivity
DMARDs: Disease-modifying anti-rheumatic drugs
ECM: Extracellular matrix
ER: Endoplasmic reticulum
ERAD: ER-associated degradation
EV: Extracellular vesicles
FasL: Fas ligand
FLS: Fibroblast-like synoviocytes
FOXP3: Forkhead box P3
GRP78: Glucose regulated protein 78 kDa
HGF: Hepatocyte growth factor
HMGB1: High-mobility group box 1
HLA: Human leukocyte antigen
Hotair: HOX transcript antisense RNA
hUCMSC: Human umbilical cord mesenchymal stem cell
ICMA-1: Intercellular adhesion molecule 1
iDCs: immature dendritic cells
IDO: Indoleamine 2,3-dioxygenase
IFN: Interferon
IL: Interleukin
IRE1: Inositol-requiring transmembrane kinase-endoribonuclease-1
JAK: Janus kinase
KLH: Keyhole limpet hemocyanin
lncRNAs: Long noncoding RNAs
LSCC: Laryngeal squamous cell carcinoma
MAMP: Mitochondrial-associated molecular pattern
M-CSF: Macrophage colony-stimulating factor
MHC: Major histocompatibility complex
MMP: Matrix metalloproteinase
MSCs: Mesenchymal stem cells
mtDNA: Mitochondrial DNA
mRNA: messenger RNA
MMP: Matrix-Metallo-Proteinase
lncRNA: Long noncoding RNA
nDNA: nuclear DNA
NF-kB: Nuclear factor kappa B
Nk: Natural killer
NO: Nitric oxide
NSAIDs: Nonsteroidal anti-inflammatory drugs
OPG: Osteoprotegerin
PAMPs: Pathogen-associated molecular patterns
PBMCs: Peripheral Blood Mononuclear Cells
PERK: Protein kinase RNA-like endoplasmic reticulum kinase
PD-L1: Programmed death-ligand 1
PGE2: Prostaglandin E2
PPI: Protein-protein interaction
PTMs: Post translation modifications
RA: Rheumatoid Arthritis
RANKL: Receptor Activator of NF-κB Ligand
RASFs: Rheumatoid arthritis synovial fibroblasts
ROS: Reactive oxygen species
SRF: Serum response factor
STA: Serum-transfer arthritis
STAT: Signal transducer and activator of transcription
TCR: T-cell receptors
TFAM: Mitochondrial transcription factor A
TGF-β: Transforming growth factor beta
Th: T-helper
Th1: T helper 1
Th17: T helper 17
TLR: Toll-like receptor
TNF: Tumor necrosis factor
Treg: Regulatory T-cell
TRAIL: TNF-related apoptosis-inducing ligand
UCMSC: Umbilical cord mesenchymal stem cell
UPR: Unfolded protein response
VCAM-I: Vascular cell adhesion molecule 1
VEGF: Vascular endothelial growth factor
WIFPWIQL: A synthetic peptide
XBP-1: X-box binding protein-1
